# Emerging Epigenetic Therapies for the Treatment of Cardiac Fibrosis

**DOI:** 10.3390/biomedicines13051170

**Published:** 2025-05-11

**Authors:** Nerea Garitano, Laura Pilar Aguado-Alvaro, Beatriz Pelacho

**Affiliations:** 1 Department of Biochemistry and Genetics, University of Navarra, 31008 Pamplona, Spain; ngaritano@unav.es (N.G.); laguado.3@unav.es (L.P.A.-A.); 2 Instituto de Investigación Sanitaria de Navarra (IdiSNA), 31008 Pamplona, Spain

**Keywords:** fibrosis, heart, epigenetic, fibroblast activation, therapies

## Abstract

Fibrosis is a pathological process characterized by excessive extracellular matrix (ECM) deposition, leading to tissue stiffening and organ dysfunction. It is a major contributor to chronic diseases affecting various organs, with limited therapeutic options available. Among the different forms of fibrosis, cardiac fibrosis is particularly relevant due to its impact on cardiovascular diseases (CVDs), which remain the leading cause of morbidity and mortality worldwide. This process is driven by activated cardiac fibroblasts (CFs), which promote ECM accumulation in response to chronic stressors. Epigenetic mechanisms, including DNA methylation, histone modifications, and chromatin remodeling, are key regulators of fibroblast activation and fibrotic gene expression. Enzymes such as DNA methyltransferases (DNMTs), histone methyltransferases (HMTs), histone acetyltransferases (HATs), and histone deacetylases (HDACs) have emerged as potential therapeutic targets, and epigenetic inhibitors have shown promise in modulating these enzymes to attenuate fibrosis by controlling fibroblast function and ECM deposition. These small-molecule compounds offer advantages such as reversibility and precise temporal control, making them attractive candidates for therapeutic intervention. This review aims to provide a comprehensive overview of the mechanisms by which epigenetic regulators influence cardiac fibrosis and examines the latest advances in preclinical epigenetic therapies. By integrating recent data from functional studies, single-cell profiling, and drug development, it highlights key molecular targets, emerging therapeutic strategies, and current limitations, offering a critical framework to guide future research and clinical translation.

## 1. Introduction

Fibrosis is a major health burden worldwide, contributing to approximately 45% of global deaths annually [1]. This high prevalence is due to the fact that fibrosis is a common consequence of numerous chronic diseases affecting different organ systems, including the heart, lungs, liver, kidneys, and skin. Among fibrotic diseases, ischemic heart disease stands out as a leading cause of mortality, responsible for nearly 9 million deaths per year [2]. Similarly, liver cirrhosis, chronic kidney disease, and interstitial lung diseases contribute significantly to global morbidity and mortality, with limited treatment options available [3]. Despite its widespread impact, fibrosis remains a poorly addressed clinical problem, with no effective therapies capable of halting or reversing its progression.

This pathological process is characterized by excessive deposition of extracellular matrix (ECM) components, primarily collagen and fibronectin, which leads to tissue stiffening and organ dysfunction [4]. Fibroblasts, the key cellular mediators of fibrosis, become activated upon injury, differentiating into myofibroblasts that produce ECM components necessary for wound healing [5]. Under physiological conditions, fibrosis is an essential repair mechanism activated in response to tissue injury. However, when the fibrotic response is exaggerated or sustained over time, ECM accumulation leads to structural damage and loss of organ function. Various molecular pathways, including transforming growth factor beta (TGF-β), platelet-derived growth factor (PDGF), and reactive oxygen species (ROS), regulate fibroblast activation and ECM remodeling across different organs [6]. Although fibrosis shares common molecular mechanisms, its clinical manifestations and progression vary significantly depending on the affected organ.

Among the different forms of fibrosis, cardiac fibrosis is particularly relevant due to its impact on cardiovascular diseases (CVDs), which remain the leading cause of morbidity and mortality worldwide. Given the critical role of fibrosis in cardiac pathology, understanding its mechanisms, classifications, and implications is essential for the development of targeted therapeutic strategies. In this review, we highlight the role of epigenetic regulation in cardiac fibrosis and discuss emerging therapeutic strategies based on epigenetic modulation. A better understanding of these mechanisms may help pave the way for more effective targeted anti-fibrotic therapies.

## 2. Classification and Progression of Cardiac Fibrosis

Cardiac fibrosis represents a key pathological process that disrupts normal heart function. This condition results from the excessive accumulation of ECM proteins, primarily collagen, within the myocardium. Unlike other tissues, the heart has a limited regenerative capacity, making fibrosis a major contributor to cardiac dysfunction and progression to heart failure (HF) [7]. Depending on its etiology and distribution, cardiac fibrosis can be classified into different types, each with distinct pathological consequences.

Reactive fibrosis occurs in response to chronic stressors such as hypertension, obesity, diabetes, or aging [8]. Unlike replacement fibrosis, reactive fibrosis is not associated with massive cardiomyocyte death. Instead, it manifests as an excessive accumulation of ECM in the interstitial and perivascular spaces, leading to increased myocardial stiffness and impaired diastolic function. In experimental models of left ventricular pressure overload, reactive fibrosis initially serves as an adaptive response to maintain cardiac structure and function. However, if the pathological stimulus persists, this process may evolve into maladaptive remodeling, ultimately leading to cardiac dysfunction and failure [9]. Moreover, reactive fibrosis is commonly observed in remote zones following myocardial infarction (MI), where it extends beyond the initial ischemic injury, further compromising cardiac function [10].

Replacement fibrosis, also referred to as reparative fibrosis, is characterized by scar formation following extensive cardiomyocyte loss, as seen in MI [11]. Due to the heart’s inability to regenerate lost cardiomyocytes, fibroblasts proliferate and deposit ECM components to preserve myocardial integrity [12]. While this process is necessary to prevent ventricular rupture and maintain structural stability, it severely impairs cardiac function by increasing tissue rigidity and disrupting electrical conduction, predisposing the heart to arrhythmias.

The progression of cardiac fibrosis occurs through a series of overlapping phases, particularly well-characterized in the context of ischemic injury, such as in MI. Following MI, the fibrotic response unfolds in three main stages: the inflammatory phase, the proliferative phase, and the maturation phase [13].

The inflammatory phase is triggered by hypoxic injury and subsequent cardiomyocyte death, which leads to the release of damage-associated molecular patterns (DAMPs) [14]. These molecules activate resident immune cells, including macrophages, as well as fibroblasts, initiating an acute inflammatory response [15]. Neutrophils rapidly infiltrate the injured myocardium, releasing ROS and proteolytic enzymes that degrade damaged tissue while amplifying inflammation [16]. Macrophages play a dual role in this phase, initially adopting a pro-inflammatory phenotype (M1) to clear necrotic debris before transitioning into an anti-inflammatory and reparative phenotype (M2) that promotes fibrosis and tissue healing [17].

The proliferative phase, occurring between days 4 and 14 post-MI, is characterized by a shift from a pro-inflammatory to an anti-inflammatory environment, allowing fibroblasts to become activated and differentiate into myofibroblasts [18]. The activation of fibroblasts is largely mediated by TGF-β and other profibrotic cytokines. These myofibroblasts migrate to the infarcted region and deposit ECM proteins, particularly type III collagen, to replace the fibrin scaffold formed during the inflammatory phase [19]. At the same time, angiogenesis is initiated, with endothelial cells (ECs) responding to hypoxic signals by forming new capillaries that facilitate oxygen delivery and tissue repair [20].

The maturation phase begins around two weeks post-MI and is marked by a transition from a loose type III collagen matrix to a more rigid and cross-linked type I collagen network [21]. Lysyl oxidase (LOX), an enzyme produced by myofibroblasts, plays a critical role in stabilizing the fibrotic scar by catalyzing collagen cross-linking [22]. Additionally, myofibroblasts progressively lose their contractile properties and transition into matrifibrocytes, a specialized fibroblast subset involved in ECM maintenance [23]. While this process ensures scar stability, it also contributes to myocardial stiffening and electrical conduction abnormalities, increasing the risk of arrhythmias and HF.

In addition to ventricular remodeling, atrial fibrosis has emerged as a relevant pathological process with important clinical implications, particularly in the context of atrial fibrillation (AF). While ventricular fibrosis is typically associated with ischemic injury, atrial fibrosis often results from chronic hemodynamic or inflammatory stress and involves endomysial collagen deposition that disrupts electrical conduction. Epigenetic dysregulation has been implicated in the pathogenesis of AF through multiple mechanisms, including altered expression of specific microRNAs, dysregulated histone acetylation, and increased activity of HMTs such as EZH2, which promotes fibrotic gene expression [24]. These findings support the concept of atrial cardiomyopathy as an epigenetically driven substrate for AF and further emphasize the importance of cardiac fibroblasts (CFs) as central mediators of fibrotic remodeling.

CFs are the primary effectors of cardiac fibrosis and play a crucial role in ECM homeostasis. Under physiological conditions, CFs maintain the structural integrity of the myocardium, regulate ECM turnover, and modulate intercellular communications with cardiomyocytes and ECs [25]. However, in response to pathological stimuli, CFs become activated, transitioning into myofibroblasts that secrete excessive ECM components, perpetuating fibrosis and impairing cardiac function [26].

Despite their essential role in cardiac fibrosis, identifying CFs remains a challenge due to their plasticity and heterogeneity. Their dynamic nature has been extensively studied using single-cell RNA sequencing (scRNA-seq), which has revealed distinct fibroblast subpopulations, their transitions, and their roles in cardiac injury and repair. Murine studies were instrumental in uncovering these diverse fibroblast states. One of the first to apply scRNA-seq in the heart was Gladka et al., who identified distinct fibroblast clusters and proposed *Ckap4* as a novel marker of activated fibroblasts following ischemia-reperfusion injury [27]. As fibroblast heterogeneity became increasingly evident, Ruiz-Villalba et al. took a step further by integrating scRNA-seq with single-cell Assay for Transposase-Accessible Chromatin-sequencing (ATAC-seq) to investigate not only transcriptional diversity but also the underlying regulatory mechanisms. Their study identified a reparative *Cthrc1⁺* fibroblast population involved in ECM remodeling and highlighted key transcription factors (TFs) such as RUNX1 and TEAD as regulators of fibroblast activation through chromatin accessibility profiling [28]. Forte et al. further expanded this work by analyzing post-MI fibroblast dynamics over time. They showed how specific fibroblast states emerged sequentially during repair, from homeostatic fibroblasts in healthy hearts to inflammatory, myofibroblastic, and late-response fibroblasts post-injury, each contributing differently to fibrosis [29]. In a broader context, Buechler et al. compared fibroblasts across tissues and disease models, identifying clusters such as *Lrrc15⁺*, *Cxcl5*⁺, and *Adamdec1*⁺ associated with fibrosis, while *Dpt*⁺ fibroblasts were linked to early activation states [30].

scRNA-seq has also been applied to human hearts, providing critical insights into disease-specific fibroblast programs. Koenig et al. profiled samples from healthy donors and patients with dilated cardiomyopathy (DCM), revealing nine fibroblast subtypes. While some were common to both conditions (Fb1, Fb2), others were enriched in DCM and linked to fibrotic remodeling (Fb5–Fb9) [31]. In a separate study, Chaffin et al. identified an activated fibroblast population expressing *POSTN*, *THBS4*, *COL1A1*, and *FAP* that was nearly absent in non-failing hearts but prevalent in both DCM and hypertrophic cardiomyopathy (HCM), underscoring disease-specific pathogenic transitions [32].

Further work by Kuppe et al. used an integrative approach combining single-nucleus RNA-seq, single-nucleus ATAC-seq, and spatial transcriptomics to define four fibroblast subtypes in post-MI human hearts. They identified a progenitor-like population (*SCARA5*⁺) in healthy regions and terminally differentiated myofibroblasts (*POSTN⁺, COL1A1*⁺) localized in fibrotic areas, driven by TFs such as TEAD and RUNX1 [33]. In the context of HF, Amrute et al. identified fibrotic subpopulations (*POSTN⁺*, *THBS4⁺*, *FAP*⁺) linked to inflammation and cytoskeletal remodeling. Recovery was marked by partial reversion to quiescence and downregulation of inflammatory and TGF-β pathways [34]. Fu et al. investigated arrhythmogenic right ventricular cardiomyopathy (ARVC), identifying fibroblast subtypes with distinct roles: FB0 and FB5 were enriched in fibrotic hearts and associated with pro-fibrotic activity, while FB3 and FB6 showed adipogenic signatures [35].

While previous studies provided detailed transcriptional and spatial maps of fibroblast diversity in cardiac fibrosis, none had addressed the epigenetic regulation underlying these cellular states. Recently, Aguado-Álvaro et al. published a preprint reporting the first large-scale functional interrogation of chromatin regulators during fibroblast transformation using combined bulk and single-cell CRISPR screens. A pooled screen in primary CFs under TGF-β stimulation identified pro- and anti-fibrotic chromatin factors, including Kat5, Wdr82, and components of the SRCAP and NSL complexes, based on their influence over fibroblast activation. To understand their role at the subpopulation level, the top regulators were further profiled using Perturb-seq under resting, fibrotic (TGF-β), and inflammatory (IL-1β) conditions. This approach revealed that specific chromatin regulators are key determinants of fibroblast plasticity, as they enable the expansion of pro-fibrotic myofibroblast subpopulations, while their depletion redirects cells toward alternative, less pathogenic or deactivated states, ultimately modulating the balance between reparative and fibrotic phenotypes in response to external cues [36].

Together, these studies not only reinforce the importance of fibroblast heterogeneity in cardiac disease progression but also underscore the need to understand how chromatin dynamics and epigenetic regulators shape fibroblast identity. This lays the foundation for exploring the role of chromatin factors in cardiovascular pathophysiology, which is discussed in the following section.

## 3. Chromatin Factors and Epigenetic Regulators in Cardiovascular Diseases

Epigenetics refers to the mechanisms that regulate gene expression without altering the DNA sequence. These mechanisms include DNA methylation, histone modifications, and chromatin remodeling, which collectively influence chromatin structure and control DNA accessibility [37]. Epigenetic modifications introduce biochemical marks that impact transcription, while chromatin remodelers reorganize nucleosomes to modulate DNA accessibility.

DNA methylation is a key epigenetic modification that involves the addition of methyl groups to cytosine residues, typically at cytosine-phosphodiester bond-guanine (CpG) dinucleotides, using DNA methyltransferases (DNMTs). This modification is generally associated with transcriptional repression, as it prevents the binding of TFs or recruits repressor complexes [38].

Histone modifications, including methylation and acetylation, regulate gene expression by altering chromatin compaction and accessibility [39]. Histone methylation is catalyzed by histone methyltransferases (HMTs), which use S-adenosylmethionine (SAM) as a methyl donor. Histone demethylation is carried out by histone demethylases (HDMs), which remove these marks and modulate gene expression [40]. Histone acetylation, catalyzed by histone acetyltransferases (HATs), relaxes chromatin and facilitates transcription, whereas histone deacetylases (HDACs) remove these marks, leading to chromatin condensation and transcriptional repression [41]. Moreover, histone acetylation on lysine residues is recognized by bromodomains (BRDs), which act as “readers” of these marks [42].

Chromatin remodeling is mediated by ATP-dependent chromatin remodelers, such as the ISWI, CHD, SWI/SNF, and INO80 complexes, which regulate nucleosome positioning, histone exchange, and chromatin accessibility [43]. These processes directly impact DNA accessibility, allowing TFs and other regulatory proteins to bind specific regions of the genome.

**Figure 1:**
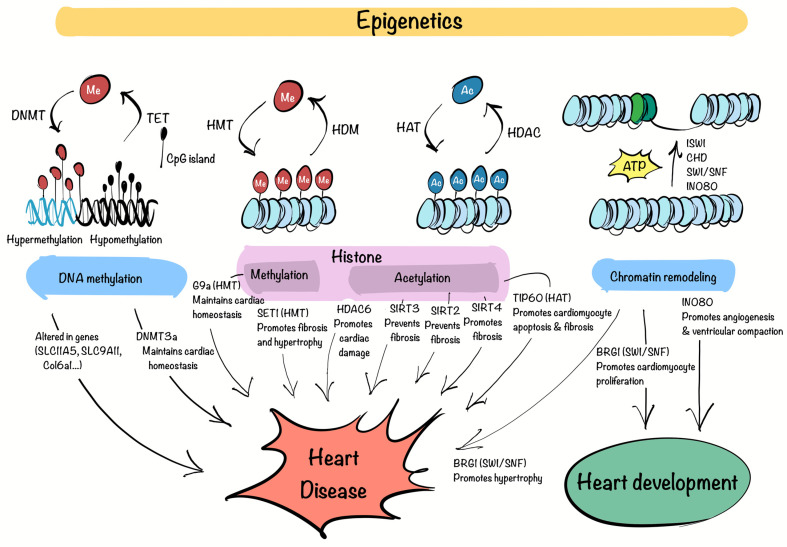
Epigenetic regulation in CVD. The complex interaction between DNA methyltransferases (DNMT), tet methylcytosine dioxygenases (TET) (DNA methylation), histone methyltransferases (HMT), histone demethylases (HDM), histone deacetylases (HDAC), histone acetyltransferases (HAT), bromodomain (Histone modifications) and ISWI, CHD, SWI/SNF, INO80 complexes (Chromatin remodeling), govern methylation (Me) of DNA and histones, acetylation (Ac) of histones, and changes in nucleosomes, consequently impacting the regulation of cardiac development, homeostasis and disease. This figure was created with Procreate (version 5.3.15).

Beyond influencing gene expression at a cellular level, these mechanisms are increasingly recognized as key contributors to cardiovascular pathology, offering insights into their potential as therapeutic targets in cardiovascular conditions (Figure 1).

DNA methylation plays a vital role in preserving genomic integrity and regulating gene expression. Disruptions in this process can lead to gene expression abnormalities associated with disease. In coronary heart disease, genes such as *SLC1A5* and *SLC9A1*, involved in glutamine metabolism and ionic balance, respectively, show altered methylation patterns, disrupting myocardial function [44,45]. Research using an MI mouse model revealed significant changes in methylation of genes like *Ptpn6*, *Csf1r*, *Col6a1*, *Cyba*, and *Map3k14*, which play key roles in disease progression. These genes may serve as biomarkers for the early detection of MI [46]. In HF, the analysis of ventricular septal tissues identified 195 regions with differential methylation, with some genes hypermethylated and others hypomethylated, impacting pathways related to cardiac remodeling, fibrosis, and mitochondrial function [47]. Additionally, a study demonstrated that DNMT3a is critical for maintaining cardiomyocyte homeostasis. Its knockout alters the expression of contractile proteins and damages mitochondrial integrity [48].

Histone modifications, such as methylation and acetylation, play a pivotal role in regulating gene expression and have been implicated in the development of CVDs. A study demonstrated that the HMT G9a is essential for maintaining cardiac function by regulating gene silencing through H3K9 dimethylation. Loss of G9a was linked to impaired cardiac contractility, increased fibrosis, and dysregulated expression of genes involved in calcium handling and hypertrophy, suggesting its potential as a therapeutic target [49]. Another example is the deletion of the H3K4 methyltransferase SET1 in a hypertensive mouse model, which reduced cardiac fibrosis and attenuated hypertrophy by modulating endothelin-1 transcription in ECs [50]. The activity of HDAC6 has been shown to worsen ischemia-reperfusion injury by increasing oxidative stress and promoting cardiomyocyte damage [51]. Conversely, SIRT3, an HDAC, protects the heart by deacetylating cyclophilin D, preventing mitochondrial dysfunction and reducing cell death [52]. In HF, a deficiency in SIRT2 has been shown to worsen myocardial fibrosis, indicating its role in counteracting fibrotic responses triggered by angiotensin II (Ang II) [53]. SIRT3, on the other hand, deacetylates GSK3β at lysine 15, enhancing its activity. This activation promotes the phosphorylation and subsequent degradation of SMAD3, thereby inhibiting fibrosis driven by TGF-β signaling [54]. In contrast, the depletion of SIRT4 has been found to reduce Ang II-induced myocardial fibrosis [55].

Among the numerous HATs, Kat5/TIP60 has recently gained attention due to its versatile and critical functions in cellular processes. TIP60 is primarily recognized for acetylating histones, but it also targets non-histone proteins, positioning itself as a key epigenetic and transcriptional regulator [56]. Building on this knowledge, researchers have begun to investigate TIP60 in cardiovascular biology, particularly given its abundant expression in myocardial tissue [57]. Recent studies have revealed its significance in ischemic heart diseases. For example, conditional deletion of *TIP60* in cardiomyocytes following MI has been associated with improved cardiac function, reduced fibrosis, and decreased apoptosis in the injured heart [58]. Another study demonstrated that TIP60 acetylates ataxia-telangiectasia mutated (ATM) protein at birth, triggering a DNA damage response (DDR) that induces cardiomyocyte senescence. Suppressing *TIP60* reduces DNA damage markers, reactivates the cell cycle, and restores a fetal-like proliferative state in cardiomyocytes, improving cardiac function after neonatal MI [59]. Furthermore, *TIP60* haploinsufficiency in adult cardiomyocytes under hypertrophic stress has been shown to enhance cell cycle activation, leading to increased proliferative activity and reduced apoptosis [60]. Building on their previous findings, the same authors investigated the long-term effects of *TIP60* depletion in cardiomyocytes. While initial results showed increased cell density and reduced apoptosis, prolonged *TIP60* loss led to progressive cardiac dysfunction, fibrosis, extensive cell death, and ultimately HF [61]. Moreover, TIP60 has also been recently identified as a pro-fibrotic factor in CFs through large-scale functional CRISPR screens, supporting its role beyond cardiomyocytes and reinforcing its relevance as a therapeutic target in fibrotic remodeling [36].

Chromatin remodelers are essential for regulating gene expression in the heart during both development and disease. Among the four main ATP-dependent chromatin remodeling families, the SWI/SNF complex has been extensively examined for its role in cardiac development. This complex, which includes the ATPase subunits BRG1 and BRM, reorganizes nucleosomes to control transcription [62]. BRG1 is critical for heart development, promoting cardiomyocyte proliferation and sustaining fetal gene expression programs [63]. Additionally, BRG1 becomes reactivated in adult cardiomyocytes under pathological conditions, where it drives the re-expression of fetal gene patterns associated with cardiac hypertrophy [64]. The INO80 chromatin remodeler also plays a pivotal role in heart development, particularly in coronary angiogenesis and ventricular compaction. Its deletion in endothelial cells impairs these processes, resulting in defective myocardial growth and congenital heart abnormalities, underscoring its importance in cardiovascular health [65].

In summary, epigenetic regulation is emerging as a crucial factor in the understanding of CVDs. The advances discussed highlight the significant role of epigenetic mechanisms in shaping cardiac health and pathology. These interconnected mechanisms influence fibroblast activation and fibrosis progression, making them promising targets for therapeutic intervention.

## 4. Epigenetic Therapies for Cardiac Fibrosis

Despite advances in understanding cardiac fibrosis, no therapy has been able to fully reverse it [66]. This challenge stems from the heart’s limited regenerative capacity, the diverse causes of fibrosis, and the heterogeneity of CFs, which complicates targeted treatments.

Current therapeutic strategies for cardiac fibrosis aim to mitigate its progression rather than completely reverse it. While these therapies do not directly target fibrosis, they play a pivotal role in reducing the factors that exacerbate its development, such as neurohormonal activation, inflammation, and hemodynamic stress. Drugs such as renin-angiotensin-aldosterone system (RAAS) inhibitors [67], including angiotensin-converting enzyme (ACE) inhibitors [68], angiotensin receptor blockers (ARBs) [69], and mineralocorticoid receptor antagonists (MRAs) [70], have shown antifibrotic effects by modulating fibroblast activation and reducing collagen deposition. Beta-blockers, commonly used in HF, also help by attenuating sympathetic overactivation, which contributes to fibrotic remodeling [71]. Statins, primarily prescribed for cholesterol control, have demonstrated pleiotropic effects in reducing oxidative stress and inflammation, indirectly influencing fibrosis progression [72].

Beyond conventional therapies, efforts to directly target fibrotic pathways have focused on key signaling mediators such as TGF-β. However, direct TGF-β inhibition has been associated with severe side effects, including increased mortality and adverse cardiac remodeling, limiting its clinical applicability [73]. Recent antifibrotic drug developments have included agents originally approved by the Food and Drug Administration (FDA) for pulmonary fibrosis, such as pirfenidone [74] and nintedanib [75]. These drugs exhibit antifibrotic properties by modulating oxidative stress, inflammation, and fibroblast activity. While promising, their efficacy in cardiac fibrosis remains under investigation, and challenges such as off-target effects and limited myocardial penetration hinder their widespread use.

Given these complexities, current therapies remain largely palliative, and in severe cases, heart transplantation is the only option, highlighting the need for novel therapeutic approaches such as epigenetic therapies [76]. Recent advancements in the understanding of epigenetic-modifying enzymes have highlighted the reversibility of many epigenetic modifications, emphasizing their role in regulating gene expression. This reversibility forms a solid foundation for developing therapeutic strategies aimed at modulating these mechanisms.

**Figure 2:**
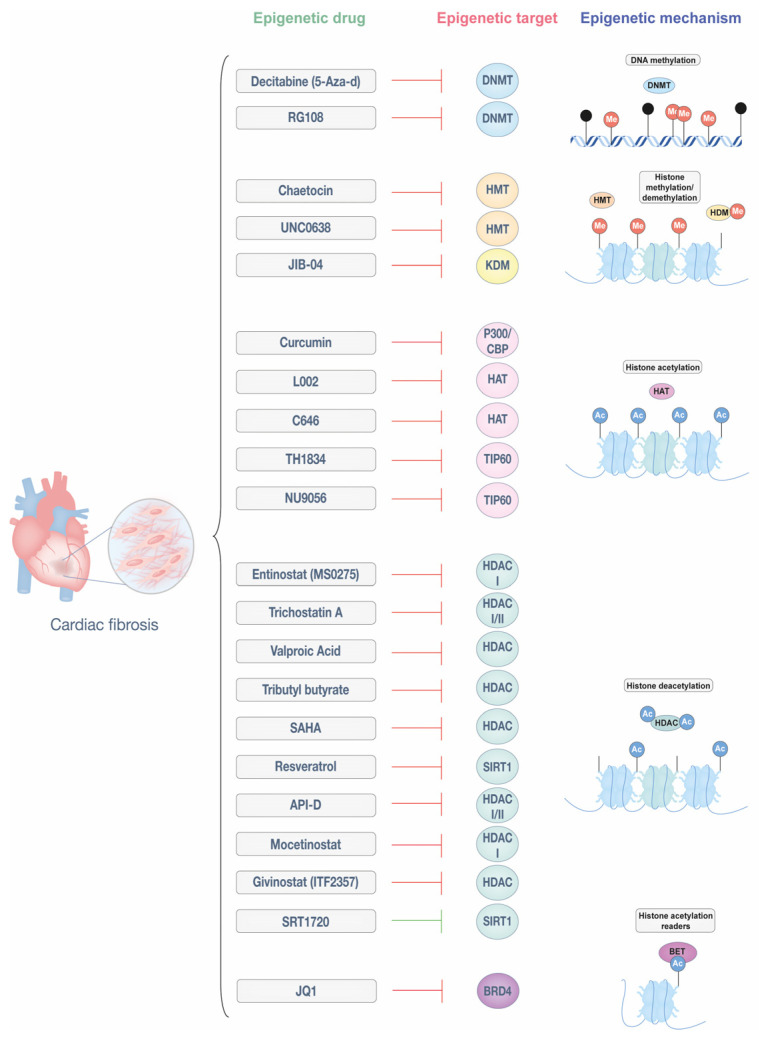
Epigenetic drugs targeting cardiac fibrosis. Epigenetic modifications regulate fibroblast activation and ECM deposition, playing a key role in the progression of cardiac fibrosis. This figure illustrates different classes of epigenetic drugs and their molecular targets, including DNA methyltransferases (DNMTs), histone methyltransferases (HMTs), histone/lysine demethylases (HDMs/KDMs), histone acetyltransferases (HATs), histone deacetylases (HDACs), bromodomains (BRD), and bromodomain and extra-terminal motif (BET) proteins. By modulating these mechanisms, epigenetic therapies hold potential for reversing or mitigating cardiac fibrosis. This figure was created with Adobe Illustrator (version 29.5).

Notably, FDA-approved epigenetic drugs, such as DNMT inhibitors (*azacitidine* and *decitabine*) and HDAC inhibitors (*vorinostat*, *romidepsin*, and *belinostat*), have proven effective in treating cancers like myelodysplastic syndrome, leukemia, and T-cell lymphomas [77]. While epigenetic therapies have primarily been explored in oncology, their application in CVDs, including cardiac fibrosis, is an area of growing interest. Although none have gained FDA approval yet for cardiovascular conditions, epigenetic inhibitors represent a promising avenue for modulating fibrosis. The potential of these drugs is illustrated in Figure 2 by their diverse targets and mechanisms, which offer innovative approaches for the treatment of cardiac fibrosis.

DNMT inhibitors, such as 5-azacytidine (also known as decitabine) and RG108, have shown potential in reducing cardiac fibrosis. For example, 5-aza-d improves cardiac remodeling and reduces fibrosis in hypertensive rats by blocking the expression of HCM genes [78,79]. Similarly, RG108 effectively prevented fibrosis and hypertrophy in mouse models of pressure overload [80,81]. While 5-aza-d integrates into DNA, potentially limiting its clinical use, small molecules like RG108 offer a more flexible approach, leveraging the reversible nature of DNA methylation to alter gene expression and promote antifibrotic effects.

Inhibitors of histone-modifying enzymes have demonstrated potential in addressing cardiac fibrosis. Chaetocin, an HMT inhibitor targeting H3K9 methyltransferase SUV39H, reduced cardiac fibrosis in a mouse model of MI [82]. UNC0638, an HMT inhibitor, reduced infarct size in an acute MI model by suppressing fibrosis, inflammation, and apoptosis through the downregulation of SMAD3 and TGF-β signaling [83]. Additionally, the demethylation of the histone mark H3K9me2 by KDM3a has been implicated in fibrosis progression, a process counteracted by the pan-KDM inhibitor JIB-04, which mitigated fibrosis in a mouse model of pressure overload [84].

Histone acetylation regulators, particularly HDAC inhibitors, are well-studied epigenetic drugs, while HAT inhibitors are emerging as promising treatments for cardiac fibrosis. Among HAT inhibitors, curcumin, a natural polyphenol with specificity for p300/CBP, reduces infarct size and cardiac fibrosis in MI models by inhibiting macrophage-fibroblast crosstalk and restraining IL18-TGFβ1-SMAD2/3 signaling. It also suppresses collagen deposition, reduces TGF-β1 production, and enhances SMAD7 expression, contributing to its antifibrotic effects [85,86,87]. Small molecule inhibitors C646 and L002 also target p300 and have been effective in attenuating hypertrophy and fibrosis in hypertension-induced HF models [88,89]. Beyond p300, the Kat5/TIP60 family has gained attention for its roles in cancer and potential in cardiac fibrosis. The experimental Kat5/TIP60 inhibitor TH1834 reduced apoptosis and fibrosis while improving systolic function in a post-MI model [90]. Similarly, another Kat5/TIP60 inhibitor, NU9056, has recently been shown to reduce fibrotic gene expression, α-SMA levels, and collagen deposition in both murine and human CFs, further supporting the therapeutic relevance of targeting Kat5 in cardiac fibrosis [36].

Despite the promising effects of curcumin and other HAT inhibitors on cardiac fibrosis, their pharmacological application remains limited by poor bioavailability, rapid metabolism, and low systemic concentrations. Curcumin, in particular, displays limited aqueous solubility and is rapidly metabolized into less active conjugates, which restricts its therapeutic efficacy after oral administration [91]. Further studies are required to enhance its bioavailability and avoid unwanted effects on other tissues or cardiac cell types. In this regard, targeted delivery systems such as polymeric nanoparticles or lipid-based carriers have emerged as optimal strategies. These nanocarriers can improve solubility, protect curcumin from premature degradation, and enable controlled release while facilitating tissue-specific accumulation, especially in fibrotic cardiac regions. Moreover, engineered nanoparticles can be functionalized to minimize systemic exposure and maximize antifibrotic activity in the heart, potentially overcoming the limitations associated with conventional administration routes [92].

HDAC inhibitors are among the most extensively studied epigenetic therapies for cardiac fibrosis, targeting different HDAC classes. Entinostat (MS-0275), a class I-specific HDAC inhibitor, reduces infarct size and improves left ventricular function after ischemia-reperfusion by decreasing oxidative damage and preserving myocardial viability. It also prevents cardiac fibrosis and mitigates electrical remodeling caused by rapid ventricular stimulation [93,94]. Similarly, Trichostatin A, an inhibitor of class I and II HDACs, prevents cardiomyocyte hypertrophy, reduces MI size, and attenuates Ang II-induced fibroblast activity by regulating the expression of matrix metalloproteinase (MMP) 9 and IL-18, enhancing its protective effects in MI [95,96,97]. Valproic acid, tributyl butyrate, and vorinostat (SAHA), all HDAC inhibitors, collectively mitigate ventricular remodeling and reduce infarct size, contributing to improved cardiac outcomes [98,99,100]. Vorinostat, in particular, has demonstrated anti-fibrotic effects across various conditions, including cardiac fibrosis. A preclinical study in an MI-induced cardiac fibrosis model showed that SAHA treatment improved cardiac function by suppressing fibrosis and reversing the activation of the TGFβ1/P38 pathway [101]. Resveratrol, a modulator of SIRT1/SMAD3 deacetylation, reduced myocardial fibrosis in a DCM model. Additionally, in the HF model, it has been shown to decrease MMP-2 activity, contributing to enhanced cardiac function and fibrosis [102,103]. API-D, a dual class I and II HDAC inhibitor, and Mocetinostat, a class I-specific inhibitor, both effectively reverse myocardial fibrosis [104,105], with Mocetinostat inducing fibroblast apoptosis and cell cycle arrest [106]. Givinostat (ITF2357) mitigates endothelial-to-mesenchymal transition and inflammation, improving heart performance and reducing fibrosis in MI models [107,108,109]. Additionally, SRT1720, a SIRT1 activator, has shown efficacy in alleviating both cardiac hypertrophy and fibrosis in a model of pressure overload. Another study demonstrated its ability to reduce fibrosis by modulating SMAD2/3 signaling [108,109].

Finally, the BRD4 inhibitor JQ1 has shown potential in reducing cardiac fibrosis by suppressing key fibrotic markers and fibroblast contractility [110,111]. A recent single-cell epigenomic study revealed that JQ1 inhibitors regulate fibroblast activation by shifting them from an activated state to a quiescent state, correlating with improved cardiac function. This effect involves the regulation of MEOX1, a TF driving fibrosis, further emphasizing JQ1’s therapeutic promise in HF and MI [111].

Overall, the exploration of epigenetic therapies has demonstrated promising results in preclinical studies, highlighting their potential to modulate key mechanisms involved in cardiac fibrosis. These findings underscore the need for further research to refine these approaches and assess their translational potential for clinical application.

## 5. Conclusions and Future Directions

In this review, we have summarized the emerging role of epigenetic therapies in cardiac fibrosis and their potential for clinical application.

Epigenetic inhibitors offer several advantages as therapeutic agents, including their reversibility, precise temporal control, and potential for clinical translation. Unlike genetic modifications, which are often permanent and associated with ethical concerns, small-molecule inhibitors allow the transient and controlled modulation of epigenetic targets. These characteristics make them particularly attractive for therapeutic applications. In cancer, epigenetic drugs have already reached clinical trials and, in some cases, received FDA approval [77]. However, the application of epigenetic therapies in CVDs is still in its early stages, and no epigenetic drug has been specifically tested in clinical trials for cardiac fibrosis.

This lack of clinical translation may be attributed to key limitations that must be addressed before these therapies can become viable treatment options. One of the major challenges in translating epigenetic therapies into clinical practice is ensuring precise delivery to the affected tissue and specific targeting of the relevant cell populations. Systemic administration of epigenetic drugs risks affecting multiple organs and cell populations, leading to off-target effects and undesirable consequences. Moreover, ensuring sufficient drug bioavailability within fibrotic myocardial tissue remains a significant hurdle.

To address these limitations, novel delivery strategies are being explored. Functionalized nanoparticles and biomaterial-based carriers have shown potential for enhancing drug accumulation in fibrotic cardiac tissue while minimizing systemic exposure. Recent studies have demonstrated that ECM-binding nanoparticles and fibroblast-specific delivery systems improve drug retention at the site of fibrosis, increasing therapeutic efficacy. For instance, MnO2-based nanozymes functionalized with tannic acid, which enhanced their affinity for ECM, have been shown to selectively accumulate in infarcted myocardium, enhancing antifibrotic effects [112]. Similarly, calcium phosphate nanoparticles modified with a cardiac-targeting peptide have enabled more efficient drug delivery via inhalation, improving ventricular remodeling and reducing fibrosis in HF models [113]. These approaches highlight the potential of targeted nanoparticle therapies to optimize epigenetic drug delivery and improve clinical outcomes.

Another promising strategy is the use of single-cell transcriptomics and epigenomics to refine the specificity of epigenetic interventions. Recent advances in single-cell technologies have revealed significant heterogeneity among CF populations, with distinct subtypes playing differential roles in fibrosis progression. Identifying and characterizing these subpopulations will allow for the development of therapies that selectively target the most pathogenic fibroblast subsets while sparing others that may contribute to tissue homeostasis.

Beyond delivery and specificity challenges, regulatory and safety considerations also limit the clinical implementation of epigenetic therapies in CVDs. One major concern is the potential risk of oncogenic activation due to global chromatin remodeling [114]. Epigenetic modifications, such as histone acetylation or methylation, can alter gene expression patterns in ways that may unintentionally activate oncogenes or suppress tumor suppressor genes. For instance, histone acetylation generally promotes gene transcription by loosening chromatin structure, which could unintentionally activate genes associated with oncogenesis. Conversely, histone deacetylation can lead to gene silencing, potentially suppressing tumor suppressor genes [115]. These off-target effects raise significant safety concerns, especially in the context of chronic treatment. Moreover, many current epigenetic drugs lack sufficient selectivity, affecting multiple pathways beyond their intended targets. This broad activity may compromise both efficacy and safety. Regulatory approval for epigenetic drugs in CVDs requires extensive preclinical and clinical evaluation and is further hindered by the absence of standardized methods to assess epigenetic changes [111]. Addressing these issues through improved drug design, delivery technologies, and biomarker-guided strategies will be essential to safely translate epigenetic therapies into the cardiovascular field.

In conclusion, the combination of emerging delivery technologies with a deeper understanding of fibroblast heterogeneity and epigenetic regulation holds promise for developing more precise and effective antifibrotic therapies. These advancements will be essential for translating epigenetic therapies into viable treatments for cardiac fibrosis, ultimately improving patient outcomes. Importantly, this review provides a comprehensive and integrative perspective that connects fibroblast heterogeneity, epigenetic regulation, and therapeutic innovation in cardiac fibrosis. By combining insights from transcriptomic and functional studies with advances in targeted epigenetic therapies, it offers a novel framework to understand disease mechanisms and guide translational research in the field.

## Data Availability

Not applicable.

## Abbreviations

The following abbreviations are used in this manuscript:

ACE	Angiotensin-converting enzyme
AF	Atrial fibrillation
Ang II	Angiotensin II
ARB	Angiotensin receptor blocker
ARVC	Arrhythmogenic right ventricular cardiomyopathy
ATAC-seq	Assay for Transposase-Accessible Chromatin-sequencing
ATM	Ataxia telangiectasia mutated
BET	Bromodomain and extra-terminal motif
BRD	Bromodomain
CF	Cardiac fibroblast
CpG	Cytosine-phosphodiester bond-guanine
CVD	Cardiovascular disease
DAMPS	Damage-associated molecular patterns
DCM	Dilated cardiomyopathy
DDR	DNA damage response
DNMT	DNA methyltransferase
ECM	Extracellular matrix
FDA	Food and Drug Administration
HAT	Histone acetyltransferase
HCM	Hypertrophic cardiomyopathy
HDAC	Histone deacetylase
HDM	Histone demethylase
HF	Heart failure
HMT	Histone methyltransferase
KDM	Lysine demethylase
LOX	Lysyl oxidase
MI	Myocardial infarction
MMP	Matrix metalloproteinase
MRA	Mineralocorticoid receptor antagonist
PDGF	Platelet-derived growth factor
RAAS	Renin-angiotensin-aldosterone system
ROS	Reactive oxygen species
SAM	S-adenosylmethionine
scRNA-seq	Single-cell RNA sequencing
TF	Transcription factor
TGF-β	Transforming growth factor beta
